# Income inequality and subjective well-being: a systematic review and meta-analysis

**DOI:** 10.1007/s11136-017-1719-x

**Published:** 2017-10-24

**Authors:** Kayonda Hubert Ngamaba, Maria Panagioti, Christopher J. Armitage

**Affiliations:** 1 Department of Social Policy and Social Work, International Centre for Mental Health Social Research, University of York, York, UK; 2 NIHR School for Primary Care Research, Manchester Academic Health Science Centre, University of Manchester, Manchester, UK; 3 Division of Psychology and Mental Health, Manchester Centre for Health Psychology, School of Health Sciences, Manchester Academic Health Science Centre and NIHR Manchester Biomedical Research Centre, University of Manchester, Oxford Road, Manchester, M13 9PL UK

**Keywords:** Subjective well-being, Happiness, Life satisfaction, Income inequality, Redistribution

## Abstract

**Background:**

Reducing income inequality is one possible approach to boost subjective well-being (SWB). Nevertheless, previous studies have reported positive, null and negative associations between income inequality and SWB.

**Objectives:**

This study reports the first systematic review and meta-analysis of the relationship between income inequality and SWB, and seeks to understand the heterogeneity in the literature.

**Methods:**

This systematic review was conducted according to guidance (PRISMA and Cochrane Handbook) and searches (between January 1980 and October 2017) were carried out using Web of Science, Medline, Embase and PsycINFO databases.

**Results:**

Thirty-nine studies were included in the review, but poor data reporting meant that only 24 studies were included in the meta-analysis. The narrative analysis of 39 studies found negative, positive and null associations between income inequality and SWB. The meta-analysis confirmed these findings. The overall association between income inequality and SWB was almost zero and not statistically significant (pooled *r* = − 0.01, 95% CI − 0.08 to 0.06; *Q* = 563.10, *I* ^2^ = 95.74%, *p* < 0.001), suggesting no association between income inequality and SWB. Subgroup analyses showed that the association between income inequality and SWB was moderated by the country economic development (i.e. developed countries: *r* = − 0.06, 95% CI −0.10 to −0.02 versus developing countries: *r* = 0.16, 95% CI 0.09–0.23). The association between income inequality and SWB was not influenced by: (a) the measure used to assess SWB, (b) geographic region, or (c) the way in which income inequality was operationalised.

**Conclusions:**

The association between income inequality and SWB is weak, complex and moderated by the country economic development.

**Electronic supplementary material:**

The online version of this article (doi:10.1007/s11136-017-1719-x) contains supplementary material, which is available to authorized users.

## Introduction

Income inequality is one of many possible determinants of subjective well-being (SWB) [1, 2]. There is a view that income inequality—the unequal distribution of household income across different participants in an economy (OECD, 2011)—is a predictor of SWB and that decreasing income inequality will boost SWB [3, 4]. However, the assumed linear relationship between income inequality and SWB is not grounded in a solid research evidence base. In fact, our scoping search yielded studies that showed mixed findings: some studies show a significant positive association between SWB and income inequality [5, 6], some show a significant negative association [4, 7, 8] and others show no significant association [9]. One explanation of these inconsistent findings is that the strength and the direction of the relationship between SWB and income inequality are moderated by other factors. For example, although both happiness and life satisfaction have been used interchangeably to assess SWB across different studies, these terms are not synonymous and might relate differently to income inequality [10]. Similarly, the literature suggests that level of economic development [11, 12], geography [8] and how income inequality is operationalised [13] may affect the relationship between income inequality and SWB [14].

Given that the relationship between income inequality and SWB is important to social policy decisions, it is surprising that no systematic evaluation of this literature has yet been undertaken. We therefore decided to undertake the first systematic review of the literature to examine the link between income inequality and SWB. The objectives were:

1. to examine the direction and the magnitude of the association between income inequality and SWB;
2. to examine the factors that may moderate the association between income inequality and SWB. On the basis of previous research evidence, we focused on the effects of
  types of measures of SWB (i.e. happiness versus life satisfaction),
  country level of development (i.e. developed countries versus developing countries),
  geographic region (e.g. studies conducted in the USA versus studies conducted in Europe).
  the way income inequality was operationalised (exogenous Gini versus endogenous Gini).

## Methods

The systematic review was conducted and reported according to PRISMA (Preferred Reporting Items for Systematic Reviews and Meta-Analyses) and Cochrane Handbook recommendations [15, 16].

### Search strategy and data sources

Systematic searches of the literature published between January 1980 and October 2017 were carried out using Web of Science, Medline, Embase and PsycINFO. Combinations of two key blocks of terms were used: (1) SWB, happiness, life satisfaction, quality of life, well-being and (2) income inequality, income level, social equality, income disparities, income redistribution. We also checked the reference lists of the studies meeting our inclusion criteria. The search strategy in each of the databases is presented in Appendix 1 (see Supplementary Material).

### Study selection

Screening was completed in two stages. Initially, the titles and abstracts of the identified studies were screened for eligibility. Next, the full texts of studies initially assessed as “relevant” for the review were retrieved and checked against our inclusion/exclusion criteria. Authors were contacted and asked for further information if necessary, most frequently for the zero-order correlation between income inequality and SWB [17]. The screening process is presented in Appendix 2 (see Supplementary Material).

### Eligibility criteria

Studies were eligible for inclusion if they met the following criteria:

1. Original studies that employed quantitative methods. Qualitative studies were excluded.
2. Included a measure of income inequality (i.e. exogenous Gini and endogenous Gini).
3. Included a measure of SWB (happiness and/or life satisfaction) [18, 19].
4. Provided quantitative data regarding the association between income inequality and SWB.
5. Were published in a peer-reviewed journal. Grey literature was excluded because they were not published through conventional and credible publishers.

### Data extraction

Information about the following characteristics of the studies was extracted: (1) first author name and year of publication, country where study was conducted, participant characteristics, period of the study, data used, research design, measures of SWB, measure of income inequality, zero-order correlations, regression coefficient, direction of the association, country level of development; and (2) methodological quality of the study, namely validity of measures, quality of the research design, population and recruitment methods, and control of confounders. Data extraction was completed by the first author. A second researcher extracted data from three randomly selected studies.

### Assessment of methodological quality

The quality review included assessment of the quality of the research design, population and recruitment methods, verified if the choice of the income inequality measure and SWB measures were valid and reliable, and if the analysis reported the association between income inequality and SWB (Table 1). Of 39 studies, 15 were given a high-quality rating of 6/6 and the remaining 24 studies were given a lower quality rating of 5/6.

**Table 1:** Included studies and quality ratings (Income inequality and SWB)

First author & year of publication	Country & participants	Period of the study	Data used	Methods analysis	SWB measures	Inc. inequality measure	Zero-order correl. *P* < 0.05	Reg. coeff., *p* < 0.05	Income inequality SWB link	Level of dev^a^	Qual. rating^b^
Alesina, 2004 US [8]	US *N* = 19,895	US-1981–1996	GSS	Ordered logit reg	Hap 1–3	Gini exogenous		US − 0.014	Gini negative ass. but sensitive to covariates (CV). Subgroups: US: Gini neg. for upper inc. group; no corr with Gini for poor and political left	Developed	6
Alesina, 2004 EUR [8]	Europe *N* = 103,773	Eur 1975–1992	Eurobarometer	Ordered logit reg	Life satisf (1–10)	Gini exogenous		EUR − 0.025	Gini negative ass. but sensitive to CV. Europe: Gini neg. for poor and political left	Developed	6
Beja, 2013 Ind [12]	14 Industrialised countries	2005	WVS	Ordinal regression	Life satisf (1–10)	Gini exogenous	− 0.0019	− 0.0003	Gini negative in both industrialised and emerging econ. but very sensitive to the industrialised econ. Both groups tolerate subjective inequality	Developed	6
Beja, 2013 Emerg [12]	19 Emerging countries	2005	WVS	Ordinal regression	Life satisf (1–10)	Gini exogenous	0.031	0.031	Gini less sensitive to emerging economies	Developing	6
Berg, 2010 [6]	Worldwide 119 countries	1993–2004	WDH	Correlation	Life satisf mood, contentment	Gini exogenous	− 0.08 (LS) mood + 0.12 cont − 0.26	+ 0.28 (CVWealth) mood + 0.28 cont. + 0.14	Life satisf. & contentment: Gini neg. at univariate level but turns positive when CV GDP in. mood: Gini positive even with CV. Subgroups: diff. in national wealth can distort. Gini neg. in Western countries, positive in Eastern Eur, Asia, Latin Am. But no sig in Africa	Worldwide	6
Blanchflower & Oswald 2004 US [30]	USA	1972–1998	GSS	Ordered logit FE	Hap	75/25 endogenous			Ineq neg. & sig, sensitive to CV; subgroups: neg. for women, low educ. Neg for US black. Higher income is associated with higher hap	Developed	5
Blanchflower & Oswald, 2004 UK [30]	UK	1973–1998	Eurobarometer	Ordered logit FE	LS	75/25 endogenous			Ineq neg. & sig, sensitive to CV; subgroups: neg. for women, low educ. Higher income is associated with higher hap; relative income matters per se	Developed	5
Bjornskov, 2013 [28]	87 countries *N* = 278,134	1990–2008	WVS	OLS	Life satisfaction (1–10)	Gini from SWIID exogenous	0.067		Subjective ineq: positive (Fairness perceptions); demand for redistribution is neg ass with SWB Gini: neg. effects of actual inequality on hap. decrease with increasing perceived fairness	Worldwide	5
Bjornskov, 2008 [31]	25 countries *N* = 25,448	1998–2004	WVS & ISSP	Ordered probit	Hap (0–10)	Gini coef. exogenous	− 0.0057		Gini neg at ind. level. But Gini positive when people believe that income distribution is ‘fair’. Redistribution can have both positive and negative effects	Developed	5
Carr, 2013 [32]	USA *N* = 9,087	1998–2008	US GSS	OLS, & multilevel	Happiness (1–3)	Gini from US census exogenous	Not provided	0.0133 (county) − 0.0762 (state)	Positive at local (county; 0.0133); Negative at State level (− 0.0762) The effect of country ineq 85% larger for high inc (− 0.2) than low-inc (− 0.375). And, the effect of state inequality on well-being is 250% larger for high incomes (0.55) than low incomes (0.22)	Developed	5
Clark, 2003 [33]	UK	1991–2002	BHPS	Ordered logit reg. FE, RE	Life satisf	Gini, 90/10 endogenous		0.104^b^ *P* < 0.10	Gini positive, sig, robust to CV. Inc ineq. seems to include some aspect of opportunity	Developed	5
Delhey & Dragolov, 2014 [34]	Europe	2007	EQLS	ML mediation	Index from Life Sat -Hap	Gini exogenous	− 0.025, − 0.029	− 0.037 (trust) − 0.023 (anxiety)	Gini neg. sig, robust to CV. Full mediation by trust, anxiety status. Distrust and status anxiety are the main explanations for the neg. effect of ineq	Developed	6
Diener, 1995 [35]	Worldwide	Diff. time points, 1984–1986	WDH	correlation	Life satisf	Gini exogenous	− 0.48	Not sig	Gini neg sig. Subgroups: Gini not sig among student sample	Worldwide	5
Dynan & Ravina, 2007 [36]	USA	1979–2004	GSS	FE reg	Hap	Gini exogenous			Hap. depend positively on how well the group is doing relative to the average in their geographic area. Robust to CV, income. People with above-average inc. are happier	Developed	5
Fahey & Smyth, 2004 [37]	Europe	1999 2000	EVS	ML OLS	Life satisf	Gini exogenous			Gini neg sig (ML) CV GDP Gini not sig (OLS)	Developed	5
Graham & Felton, 2006 [38]	Latin America	1997–2004	Latino Baromet	Ordered logit cluster	Life satisf	Gini exogenous			Ineq. has negative effects on happiness in Latin America (LA). But Gini not sig. when control for wealth. Ineq. or relative position matters more in LA	Developing	5
Grosfeld, 2010 [39]	Poland *N* = 1081–3168	1992–2005	Poland CBOS	Ordered logit	Satisfaction with country economy (1–5)	Gini(endogenous)^b^	0.074	0.087	Positive, then neg when expectation change	Developed	6
Gruen, 2012 [40]	21 Transition countries (TC) in Europe	1988–2008	WVS	Regression analysis	Life Satisfaction (1–10)	Gini from SWIID	− 0.132		No significant when all, but negative in TC. No significant in TC in the last wave	Developed	6
Hagerty, 2000 [29]	USA	1989–1996	GSS	OLS	Hap	80/20 Pareto principle			Neg sig for 80; positive sig for 20; not sig for mean income	Developed	5
Hajdu, 2014 [41]	29 EU Countries *N* = 179,273	2002–2008	ESS	OLS regressions	Life satisfaction (0–10)	Gini from SWIID	− 0.045	− 0.036	People in Europe are negatively affected by income inequality, whereas reduction of inequality has a positive effect on well-being. a 1% point increase in the Gini index results in a − 0.036 point lower satisfaction	Developed	6
Haller & Hadler, 2006 [42]	Worldwide	1995–1997	WVS	ML	Life satisf, Hap	Gini			Gini positive sig. Subgroups: Latin America : high inc ineq but happier; Eastern Europe: high inc ineq & less happy	Worldwide	5
Helliwell, 2003 [43]	Worldwide	1980–1997	WVS	OLS FE	Life satisf	Gini			Gini not sig	Worldwide	5
Helliwell & Huang, 2008 [44]	Worldwide	1980–2002	WVS/ EVS	OLS, Correl	Life satisf	Gini			Gini positive sig robust to CV. Subgroups: Gini positive in Latin America, poorer countries & poor governance nations	Worldwide	5
Jiang, 2012 [45]	China *N* = 5630	2002	CHIP	OLS; ANOVA	Happiness (1–5)	Gini (endogenous)^b^	Not provided		Positive when they look local BUT Negative with between group inequalities	Developing	5
Knigh, 2010 [46]	China *N* = 6813 in urban *N* = 9160 in rural	2002	CHIP	OLS	Happiness (1–5)	Gini (lowest, middle, highest)^b^	Not provided		Change with reference group. Positive at county level. Urban less happier than rural	Developing	5
Layte, 2012 [47]	Europe	2007/ 2008	EQLS	ML	WHO5 Hap	Gini			Gini neg sig, sensitive to CV. Subgroups: Gini effect stronger in high inc. countries	Developed	5
Lin, 2013 [48]	116 countries	2006	WH & Country mean	OLS & SAR	Happiness (0–10)	Gini (equal < 40 & unequal > 40)	− 0.23		Importance of group clustering in the studies of hap. Unemp high in unequal soc Better governance, equal opport. improve hap	Worldwide	5
Morawetz, 1977 [49]	Israel	1976	–	Correlations	Hap	Equal/unequal			Equal societies happier and Unequal societies less happy	Developed	6
Ngamaba, 2016 [50]	Rwanda	2007 & 2012	WVS	ML FE	Hap 1–4 LS 1–10	Gini from SWIID	Hap 0.269 LS 0.371	Not provided	In Rwanda: Gini positive sig, sensitive to CV. When all nations are included: the positive Gini (Hap 0.071, LS 0.043) change to negative (Hap − 0.031, LS -0.039), sensitive to CV	Developing	6
Oishi, 2011 [4]	USA *N* = 53,043	1972–2008	US GSS	Multilevel mediation	Happiness (1–3)	Gini from US census	− 0.37	− 0.206	Negative, mediated by fairness and trust	Developed	6
Oishi, 2015 HIC [51]	16 countries (high income nations)	1959–2006	Veenh. World database of hap	Multilevel	Different measures, also LS (1–4)	Gini from UNU-WIDER	− 0.022	−0.022	Negative after controlling for GDP per capita	Developed	6
Oishi, 2015 Latin Am [51]	18 Latin American Countries	2003–2009	Latinobarometro data	Multilevel	Life satisf (1–4)	Gini from the World Bank	− 0.005 *P* = 0.067	− 0.007 *p* = 0.010	Negative after controlling for GDP per capita. Some authors may argued that these findings are close to 0 and no sig (− 0.005, P = 0.067)	Developing	6
Rozer, 2013 [5]	85 Countries *N* = 195,091	1989–2008	WVS	OLS, Multilevel	Index from LS (1–10) & Hap (1–4)	Gini (exogenous)	0.04		Positive, weaker when people trust more others	Worldwide	5
Schwarze and Harpfer, 2007 [52]	West Germany	1985–1998	Socio Econ Panel	OLS	Life satisf	Atkinson inequality measure			Gini neg sig	Developed	5
Senik, 2004 [53]	Russia *N* = 4685	1994–2000	RLMS	Ordered probit	Life Satisfaction	Gini from reference group income	0.331		Gini Positive, total effect: Gini not sig. Support the “tunnel effect”. The ref group’s income exerts a positive influence on individual LS	Developing	5
Tao, 2013 [54]	Taiwan *N* = 1277	2001	TSCS	OLS & Ordered probit	Happiness (1–4)	Gini (endogenous) rich, middle, poor	Not provided		Negative but change to positive when perception on reference group change	Developed	5
Wang. 2015 [55]	China N = 8,208	2006	CGSS	Ordered probit model	Hap (1–5)	Gini	− 0.0382		Ind. hap. increases with Gini when Gini is < 0.405. Then decreases when 60% of the pop have > 0.405	Developing	5
Verme, 2011 [14]	84 countries N = 267,870	1981–2004	WVS & EVS	Ordered logit	Life Satisf (1–10)	Gini WVS	− 0.029		Gini neg and sig on LS. Robust across dif. inc. groups and countries. Sensitive to multicollinearity generated by the use of country and year fixed effects, and if Gini data points is small. Subgps Poor: − 0.023; No poor: − 0.031; Western: − 0.035; no Western: − 0.016	Worldwide	5
Zagorski, 2014 LS [9]	28 EU *N* = 20, 498–26,257	2003	EQL	Multilevel	Life sat. (1–10) Hap (1–10)	Gini	LS − 0.19 Hap − 0.14	− 0.03 no sig	No sig.; income inequality does not reduce SWB in advanced societies	Developed	6

*SWB* subjective well-being, *BHPS* British Household Panel Survey, *NSCW* National Study of the Changing Workforce, *WVS* World Value Survey, *GSS* General Social Survey, *ISSP* International Social Survey Programme, *CHIP* Chinese Household Income Project, *WDH* World database of Happiness, *RLMS* Russian Longitudinal Monitoring Survey, *CBOS*: Polish Public Opinion Research Center, *TSCS* Taiwan Social Change Survey, *WIDER* World Institute for Development Economics Research, *EQL* European quality of life, *CGSS* China General Social Survey, *ESS* European Social Survey, “*Hap* 1–4” means the study assessed Happiness on a 1–4 scale; “*LS 1–5*” means the study assessed life satisfaction on a 1–5 scale, *OLS* Ordinary Least Squares, *SAR* Spatial autoregressive, *CV* covariates, *sig* significant, *Dev* development

^a^We classified country level of development according to the World Bank estimate [20]

^b^The quality assessment score is calculated by awarding 1 point for each of the criteria such as valid recruitment procedure, research design, income inequality measures, SWB measures and if the outcome of the association was reported

### Narrative synthesis

The narrative synthesis of all 39 eligible studies focused on the way SWB is assessed, country level of development, geographic region and the way income inequality was operationalised.

### Data analysis

Our plan was to pool the results of the association between income inequality and SWB across the individual studies using meta-analysis. Authors of published papers that did not report data in a form amenable for meta-analysis were contacted and eight authors provided further information. We performed a meta-analysis on all 24 studies reporting the correlation coefficients between income inequality and SWB. Studies that assessed both happiness and life satisfaction were reported separately in the subgroups in order to test whether variation is due to the way SWB was assessed. Using the World Bank classification of countries, we performed another subgroup analysis to examine whether the results differed between developed and developing countries. According to the World Bank, developed countries are defined as industrial countries, advanced economies with high level of Gross National Income (GNI) per capita of 12,736 US dollars per year (estimated in July 2015) [20, 21]. In contrast, developing countries includes countries with low and middle levels of GNI per capita (> 12,736 US dollars) [20, 21].

The associated Confidence Intervals (CI) of the zero-order correlations were calculated in STATA 13.1 [22]. The pooled zero-order correlation as well as the forest plots were computed using the meta-an command for STATA [22]. A random effects model was used for all the meta-analyses because of anticipated heterogeneity. Heterogeneity was assessed using the Cochran’s Q and Higgin’s I^2^ statistic [16]. We focus our interpretation of the results in terms of effect sizes [23]. To test whether the association between income inequality and SWB varies across subgroups, we used Cohen’s q to test whether there were significant differences in the magnitudes of the correlation coefficients following Fisher’s z transformation of *r* [24]. By convention, if *z* score values are greater than or equal to 1.96 or less than or equal to − 1.96, the two correlation coefficients are significantly different at a 0.05 alpha level (suggesting difference of correlation coefficients between two population groups) [25, 26].

## Results

**Fig. 1:**
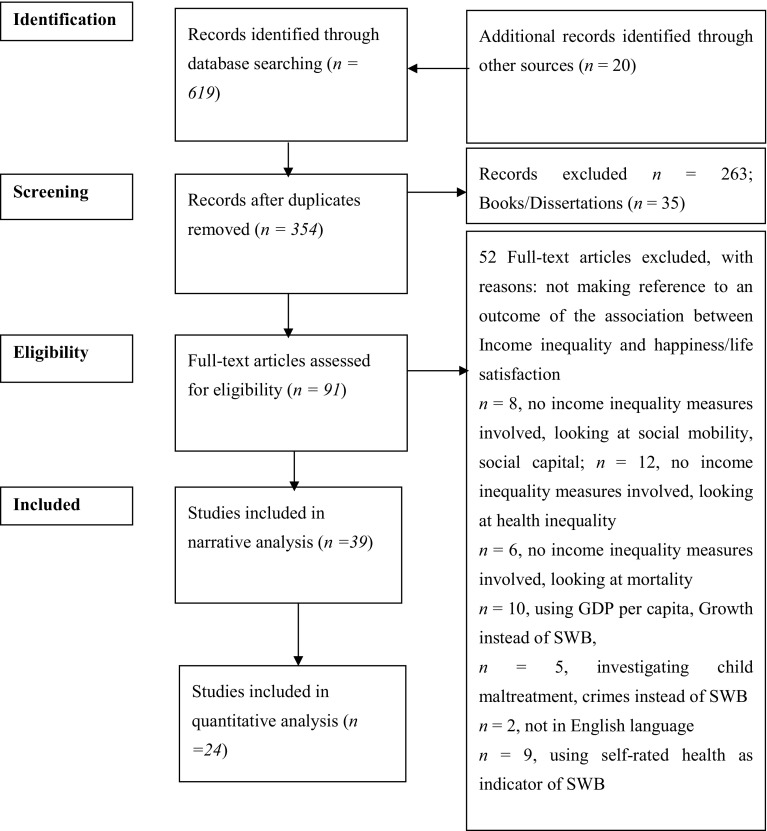
PRISMA flow diagram (income inequality and SWB); source: [15, 27]

A total of 619 titles were retrieved, and after removing duplicates (*n* = 250), 336 journal articles, 30 books and 5 dissertations were screened for relevance. Following tittle/abstract and full-text screening, 39 articles were deemed eligible for the narrative analysis and 24 studies were eligible for meta-analysis. The flowchart of the screening and selection process is shown in Fig. 1.

### Descriptive characteristics of the studies

Table 1 presents the main characteristics of the 39 articles included in the review. Table 1 provides details about the country in which each study was conducted, participant characteristics, data used, research design and measures used to assess SWB and income inequality. Table 1 presents the zero-order correlation and regression coefficients, the outcome of the association between income inequality and SWB, and the quality ratings.

Six studies were conducted in the USA, 11 studies were conducted in Europe, two in Latin America, ten worldwide (including all continents) and nine elsewhere or used different groupings (e.g. three in China, two in Industrialised countries, one in Russia, one in Israel, one in developing countries and one in Taiwan)—please see Table 1 for more details. All studies were published between 1977 and 2015 and participants were adults aged between 16 and 99 years. The sample size varied from 1277 to 278,134 and recruited from different groups including students, workers, self-employed and general population. Studies used data from a range of surveys such as the General Social Survey (GSS), World Value Survey (WVS), Eurobarometer, world database of happiness (WDH), European quality of life (EQL) and Chinese Household Income Project (CHIP). Most studies were conducted in developed nations. Only four studies were conducted exclusively in developing countries (three studies in China and one study in Russia). Different measures were used to assess SWB (e.g. happiness [4] and life satisfaction [12]) and income inequality (e.g. Gini coefficient [28], 80/20 skew [29]).

### Narrative synthesis of the results including studies with non-amenable data

Thirty-nine studies were included for the narrative analysis of the association between income inequality and SWB. The overall evidence for the relationship between income inequality and SWB was mixed, negative, positive or non-significant across studies (see Table 1). The narrative synthesis focused on four factors.

#### SWB assessment (i.e. happiness versus life satisfaction)

14/39 studies assessed happiness and 21 studies used life satisfaction to assess SWB. The remaining four studies used both happiness and life satisfaction to assess SWB. Of 14 studies using happiness to assess the SWB, eight reported a negative association and six reported a positive association with income inequality. Of 21 studies using life satisfaction to assess SWB, 12 reported a negative association, six reported a positive association and three found no relationship. The remaining four studies that used both happiness and life satisfaction reported negative (*n* = 2), positive (*n* = 1) and no (*n* = 1) associations.

#### Country level of development

Using the World Bank classification of countries [20], our narrative analysis shows that 21 studies were conducted in developed countries, of which 18 reported a statistically significant negative association between income inequality and SWB and the remaining three report a statistically significant positive association. Studies that were conducted worldwide (*n* = 9) report both negative (*n* = 4) and positive (*n* = 4) associations, and one study found no association [44]. The remaining nine studies that were conducted in developing countries report a positive (*n* = 6) or no association (*n* = 3) between income inequality and SWB. Studies conducted in Russia, rural China and Rwanda report a positive association between income inequality and SWB [46, 50, 53, 56]. While all three countries are classified as developing countries, their GDP per capita varied considerably from $9092 in Russia to $8027 in China and $697 in Rwanda [100].

#### Geographic region

Of 39 studies, one study (i.e. Alesina and colleagues) compared Europeans to Americans [8] and found that the association between income inequality and SWB was stronger among Europeans than Americans. A cross-national study investigating the association between income inequality and SWB in 119 nations reported mixed findings: a negative association in the Western world (i.e. Western European countries, US, Canada, Australia and New Zealand); a slightly positive association in Eastern Europe, Asia and Latin America (after controlling for wealth) and no association in Africa [6]. Berg and Veenhoven [6] reported only the overall association and did not report the quantitative data supporting the negative association in Western countries or either the positive or no association in other regions [6].

#### The way income inequality was operationalised (i.e. exogenous Gini and endogenous Gini)

The majority of studies (*n* = 26) used exogenous Gini (i.e. extracted from nation-level data) and the remaining 13 studies used endogenous Gini (i.e. calculated from individuals’ responses). Studies that used endogenous Gini were longitudinal studies and conducted in single countries such as the UK, Russia, China and Poland, whereas studies using exogenous Gini (*n* = 18) were mainly cross-sectional. In both groups, the studies have reported both negative and positive associations between income inequality and SWB regardless of whether the Gini coefficient was exogenous or endogenous.

### Meta-analysis of the association between income inequality and SWB

#### Overall relationship between income inequality and SWB

**Fig. 2:**
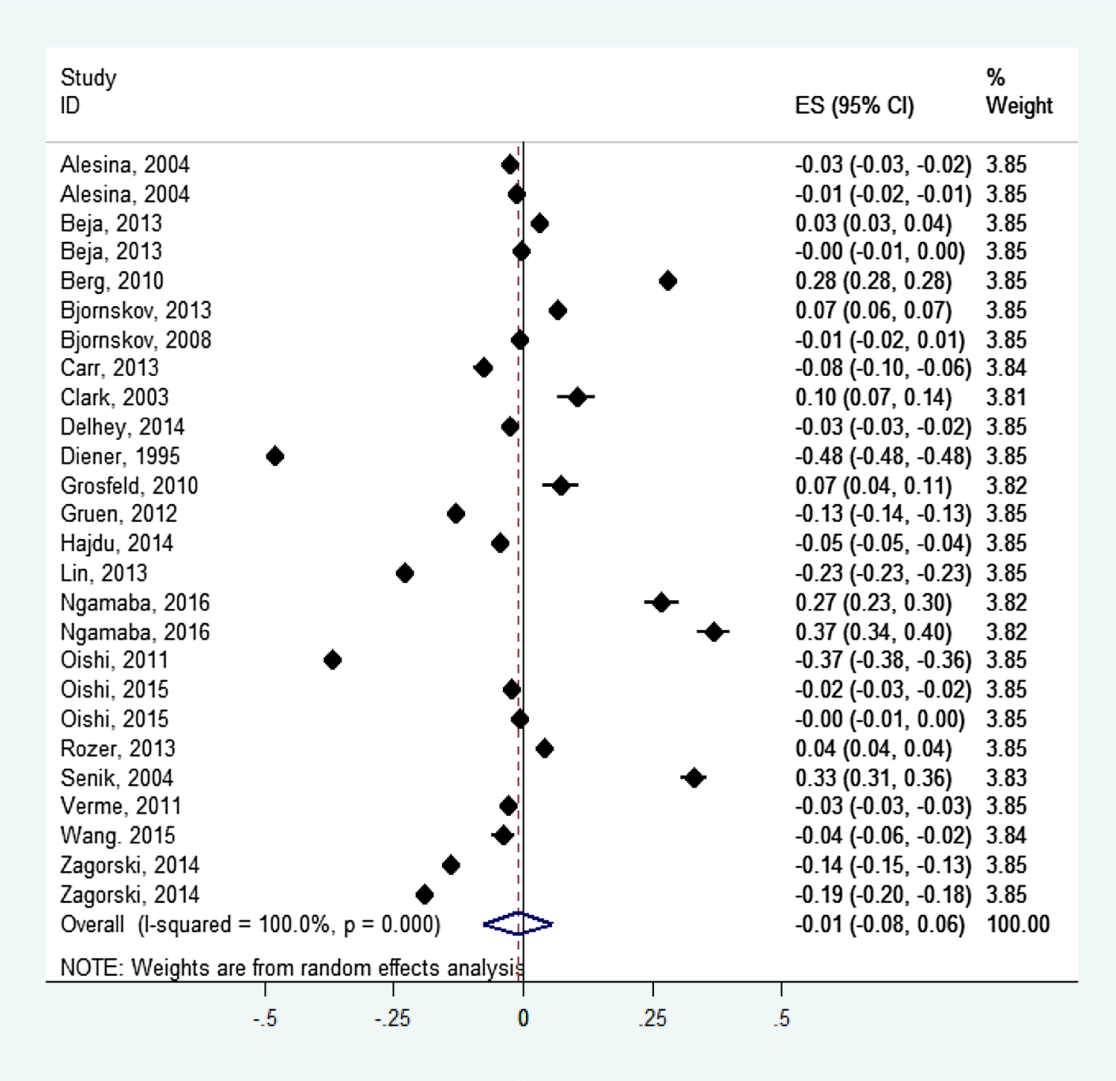
Forest plot displaying meta-analysis of the correlations between income inequality and SWB across 24 independent samples

Figure 2 presents the forest plot of the main analysis, namely the overall relationship between income inequality and SWB across the 24 studies that provided the relevant statistics. The overall pooled effect size was practically zero and non-significant, suggesting that there is no association between income inequality and SWB (pooled *r* = 0.01, 95% CI − 0.08 to 0.06) and the heterogeneity between studies was high (*Q* = 563.10, *I* ^*2*^ = 95.74%, *p* < 0.001). As shown in Fig. 2, the effect sizes of the individual studies included in the meta-analysis differed considerably in direction and magnitude. Sixteen studies reported a negative association between income inequality and SWB, whereas eight studies reported a positive association between income inequality and SWB.

#### Results of the subgroup analysis

##### Country level of development

Of 24 studies eligible for the meta-analysis, 14 studies were conducted in developed countries (e.g. USA) versus five studies conducted in developing countries (e.g. China). The pooled effect sizes across studies based on populations from developed and developing countries were statistically significant in both groups indicating that the relationship between income inequality and SWB does differ across developed and developing countries (developed countries: pooled *r* = − 0.06, 95% CI − 0.10 to − 0.02; developing countries: pooled *r* = 0.16, 95% CI 0.09–0.23). The results of the Cohen’s *Q* test confirmed that the magnitude of the correlation was significantly negative among studies conducted in developed countries and significantly positive among studies conducted in developing countries: Cohen’s *q* = 24.556, *p* < 0.05 (See Fig. 3 in Appendix 3 (Supplementary Material)).

##### Geographic region (USA vs. European countries)

Of 24 studies eligible for the meta-analysis, three studies were conducted in the USA versus seven studies conducted in European countries. The pooled effect sizes in these two regions (i.e. studies conducted in the European countries and the USA) were statistically significant indicating a negative association between income inequality and SWB (European countries: pooled *r* = 0.05, 95% CI − 0.09 to − 0.01; USA pooled *r* = − 0.08, 95% CI − 0.14 to − 0.01) (See Fig. 4 in Appendix 3 Supplementary Material).

##### SWB measures

The meta-analysis involved eight studies that used happiness to assess SWB versus 18 studies that used life satisfaction to assess SWB. The main effect was not influenced by type of SWB measures (life satisfaction: pooled *r* = 0.02, 95% CI − 0.06 to 0.10; happiness: pooled *r* = − 0.08, 95% CI − 0.18 to 0.03) (See Fig. 5 in Appendix 3 (Supplementary Material)).

##### Exogenous Gini versus endogenous Gini

Of 24 studies eligible for the meta-analysis, the majority of studies (*n* = 18) used exogenous Gini, while the remaining six studies used endogenous Gini. The pooled effect sizes between studies that used exogenous Gini and studies that used endogenous Gini were statistically non-significant indicating that the relationship between income inequality and SWB does not vary when exogenous or endogenous Gini was used (exogenous Gini: pooled *r* = − 0.02, 95% CI − 0.10 to 0.06; endogenous Gini: pooled *r* = 0.03, 95% CI − 0.09 to 0.16) (See Fig. 6 in Appendix 3 (Supplementary Material).

## Discussion

The association between income inequality and SWB is complex and highly dependent on methodological variations across studies. The findings of this review do not support a link between income inequality and SWB in general. Subgroup analyses revealed that the association between income inequality and SWB is significantly influenced by the country economic development. The association between income inequality and SWB is significantly negative in developed countries (pooled *r* = − 0.06, 95% CI − 0.10 to − 0.02) but significantly positive in developing countries (pooled *r* = 0.16, 95% CI 0.09–0.23).

Nevertheless, the association between income inequality and SWB was not influenced by (a) the measure used to assess SWB (i.e. happiness and life satisfaction), (b) geographic region (i.e. studies conducted in the USA versus studies conducted in the European countries) or (c) the way income inequality was operationalised (i.e. exogenous Gini vs. endogenous Gini).

### How to interpret the exploratory findings?

Our findings suggest that the direction of the association between income inequality and SWB differs between developed and developing countries. Differences in different preferences for income inequality might explain this finding. For example, the evolutionary modernisation theory [11, 58] hypothesises differences in tolerance for income inequality as economies move from developing to developed countries. According to this theory [11, 58], people in developing countries might perceive income inequality as an economic opportunity or incentive to work, innovate and develop new technologies and therefore as a more core determinant of their well-being compared to developed countries. In contrast, technology, economic growth and innovation might be taken for granted in developed countries, meaning that income inequality may be perceived as a treat rather than a challenge [11, 59]. Moreover, our findings do support the “tunnel” effect theory suggesting that the rise of income inequality may signal future mobility and an increase of SWB [60]. The “tunnel” effect theory supports the idea that people in developing countries may tolerate income inequality by observing other people’s increasingly rapid progression and interpreting this evolution as a sign that their turn will come soon [60, 61]. A study conducted in Poland found that when an increase of income inequality was associated with growth and when it was perceived to change rapidly, people were more satisfied with their lives [39]. For example, Berg has suggested that “income inequality is not necessary harmful to well-being. Beja added that people may accept income inequality when they see the possibilities to rise above their current position” ([12], p. 153).

### Research and social policy implications

The main contribution of this systematic review and meta-analysis is that the country level of development influences the link between income inequality and SWB: income inequality is more likely to be a contributor to SWB in citizens of developing countries than in developed countries. Reducing income inequality could be a potentially fruitful approach for governments and policy makers of developed countries as a means of improving the SWB of their citizens [11, 12]. The inverse association of SWB with income inequality in developing countries suggests that income inequality is more likely to be seen as job opportunities for innovation in these countries. However, this review was only based on cross-sectional studies and no causal inferences are allowed; longitudinal studies are needed prior to forming any causal links. The association between income inequality and SWB was not influenced by the measure used to assess SWB, geographic region or the way income inequality was operationalised. Our findings are in line with previous research conducted in OECD countries suggesting no association between income inequality and SWB [9] “the best evidence that we have to date is that redistribution beyond the minimum for advanced societies does not enhance subjective well-being/quality of life” ([9], p. 1107). Nevertheless, further studies are needed to understand the circumstances in which income inequality reduces SWB [3, 4, 62] versus the circumstances in which income inequality is not necessarily harmful to SWB [6, 12]. For example, extraordinary circumstances such as the great recession may affect how inequality is associated to subjective well-being. This gap in knowledge is critical because some government and policy makers still ask whether people care about income inequality and if income inequality affects SWB. At present, the evidence base is weak and cannot support strongly such decisions. Most importantly, the present systematic review highlights the need to produce a higher-quality evidence base to support social and political decisions relating to income inequality and SWB, both with respect to identifying (a) what are the consequences of income inequality and (b) what are the antecedents of SWB.

### Strengths and limitations

This review has several strengths. First, the search was conducted according to PRISMA published guidance [27]. Consistent with the Cochrane guidance [16], the search strategy comprised a thorough literature review, screening of reference lists and contacting authors for additional information. Second, this is the first systematic review that investigated the association between income inequality and SWB, and therefore the findings of this review have the potential to inform the literature in this area.

Nevertheless, it is important to recognise few key limitations of this review. First, the preponderance of cross-sectional studies means that it was impossible to establish a temporal or causal relationship between income inequality and SWB. Second, the poor reporting of data in combination with the use of different analytic approaches precluded any firm conclusions about the direction and strength of the association between income inequality and SWB. Future studies are encouraged to concentrate on establishing an initial correlation between income inequality and SWB before embarking on multivariate analyses. Third, this study investigated the relationship between income inequality and SWB. Nevertheless, previous studies investigating people’s quality of life have reported a link between inequality, SWB and health status [5, 9]. Further studies are needed to systematically investigate the association between income inequality, SWB and health status [1, 2]. Finally, the majority of studies included in this review were conducted in developed countries (*N* = 14) and only five studies were conducted in developing countries. This is problematic in terms of the representativeness for the purpose of global decision-making. More studies are needed to be performed in developing countries. Due to limitations in the available data, we were unable to compare Latin America to Europe or the USA because only one Latin America country had data amenable to meta-analysis. Social and political history may affect the association between income inequality and SWB because Inglehart et al. report that, with the same level of wealth, Latin America is happier than their counterparts in Ex-Communist nations [59]. We strongly encourage more methodologically sound investigations to examine the association between income inequality and SWB and to elucidate current gaps and inconsistencies.

### Conclusion

In conclusion, this is the first systematic synthesis of the literature regarding the link between income inequality and SWB. The main finding of this review is that the association between income inequality and SWB is complex. More rigorous investigations are needed to elucidate the link between income inequality and SWB, and to identify what are the antecedents and consequences of income inequality and SWB taking into account the country development level.

## Electronic supplementary material

Below is the link to the electronic supplementary material.

Supplementary material 1 (DOCX 46 KB)

Supplementary material 2 (DOCX 43 KB)

Supplementary material 3 (DOCX 8714 KB)
