# Supplementary material for: Income inequality and subjective well-being: a systematic review and meta-analysis
Source: Qual Life Res. 2017 Oct 24;27(3):577–96. doi: 10.1007/s11136-017-1719-x (PMC5845600; doi:10.1007/s11136-017-1719-x)
Supplement: Supplementary file 1 — Supplementary material 1 (DOCX 46 KB) [file 11136_2017_1719_MOESM1_ESM.docx]

**Appendix 1: Search terms and strategies used**

1. Happiness OR life satisfaction OR subjective well-being OR quality of life

AND

1. Income inequalit* OR income disparit* OR income deprivation

We did use three steps in each database

The 1^st^ step was to look at subjective well-being using these words: Happiness OR life satisfaction OR subjective well-being OR quality of life

The 2^nd^ step was to look at income inequality using these words: Income inequalit* OR income disparit* OR income deprivation.

We used terms such as: Gini coefficient, pareto principle 80/20, the Atkinson inequality measures, Coefficient of variation and Robin Hood index.

The 3^rd^ step was to look at both using AND between these two

Note: for web of science, we have used

Subjective NEAR/2 well* being OR life NEAR/2 satisfaction OR quality NEAR/2 life OR happiness

Income NEAR/2 inequalit* OR income NEAR/2 disparit* OR income NEAR/2 deprivation

**Step 1**

Web of Science

663,783

Embase

319,405

PsycINFO

64,731

Medline

129

**Step 2**

Embase

985

Web of Science

14,759

PsycINFO

713

Medline

30

**Step 3**

Web of Science

453

Embase

33

PsycINFO

59

Medline

10

**After deleting duplication**

Web of Science

252

Embase

33

PsycINFO

59

Medline

10
