# Supplementary material for: Income inequality and subjective well-being: a systematic review and meta-analysis
Source: Qual Life Res. 2017 Oct 24;27(3):577–96. doi: 10.1007/s11136-017-1719-x (PMC5845600; doi:10.1007/s11136-017-1719-x)
Supplement: Supplementary file 2 — Supplementary material 2 (DOCX 43 KB) [file 11136_2017_1719_MOESM2_ESM.docx]

**Appendix 2: Screening Process (Income inequality and SWB)**

Web of science

Review 25% for homogeneity

Rejected by 2 reviewers

Accepted by 2 reviewers

Accepted by 1 of 2 reviewers

EXCLUDE

Medline

PsycINF0OCINAHL

EMBASE

Create database

Identify duplicates

Original articles

SCREENING (titles/abstracts)

Rejected by 2 reviewers

Accepted by at least 1 reviewer

Review 10% for homogeneity

SCREENING (full text)

EXCLUDE

**INCLUDE**

Discussion until consensus achieved

EXCLUDE
