# Supplementary material for: Income inequality and subjective well-being: a systematic review and meta-analysis
Source: Qual Life Res. 2017 Oct 24;27(3):577–96. doi: 10.1007/s11136-017-1719-x (PMC5845600; doi:10.1007/s11136-017-1719-x)
Supplement: Supplementary file 3 — Supplementary material 3 (DOCX 8714 KB) [file 11136_2017_1719_MOESM3_ESM.docx]

Appendix 3


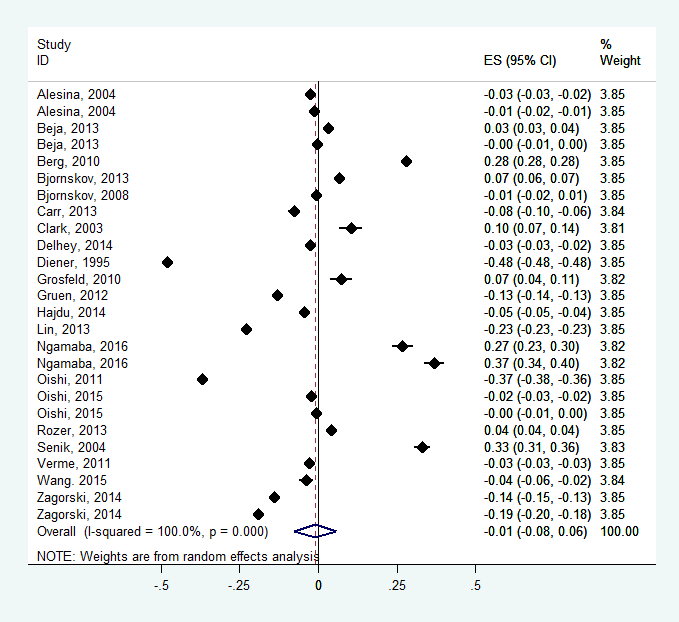


Figure 2. Forest plot displaying meta-analysis of the correlations between income inequality and SWB across 24 independent samples.


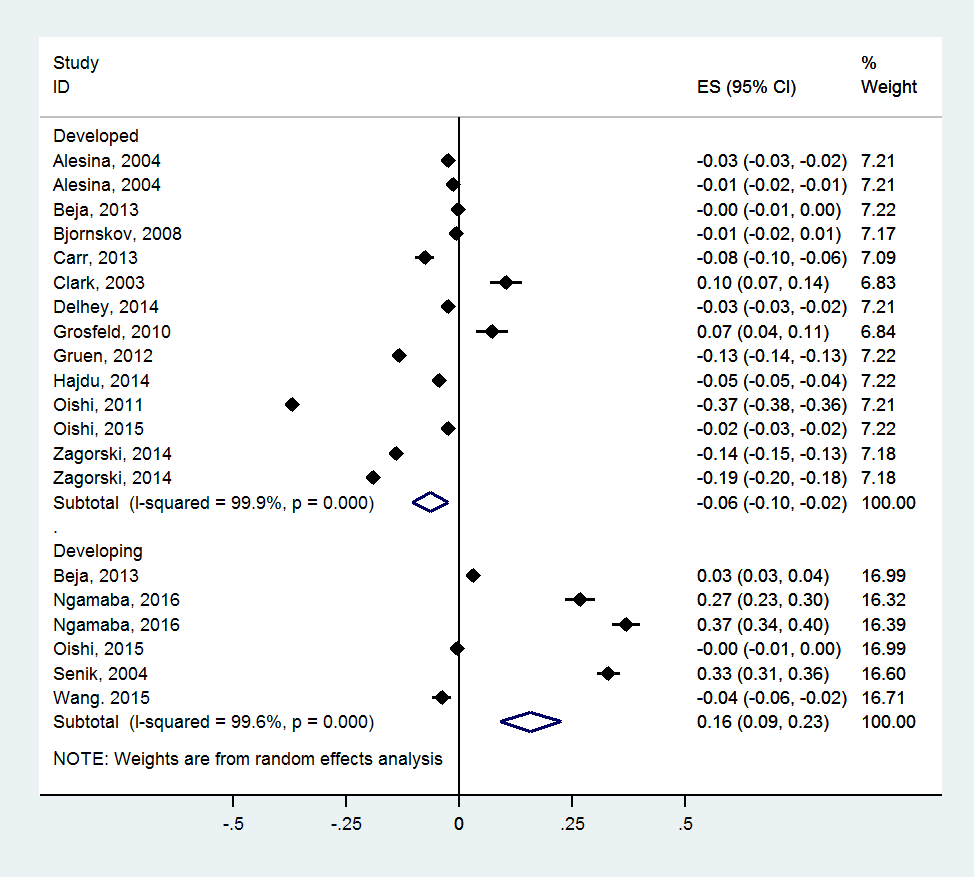


Figure 3. Forest plot displaying meta-analysis of the correlations between income inequality and SWB of sub-group: Developed versus Developing countries


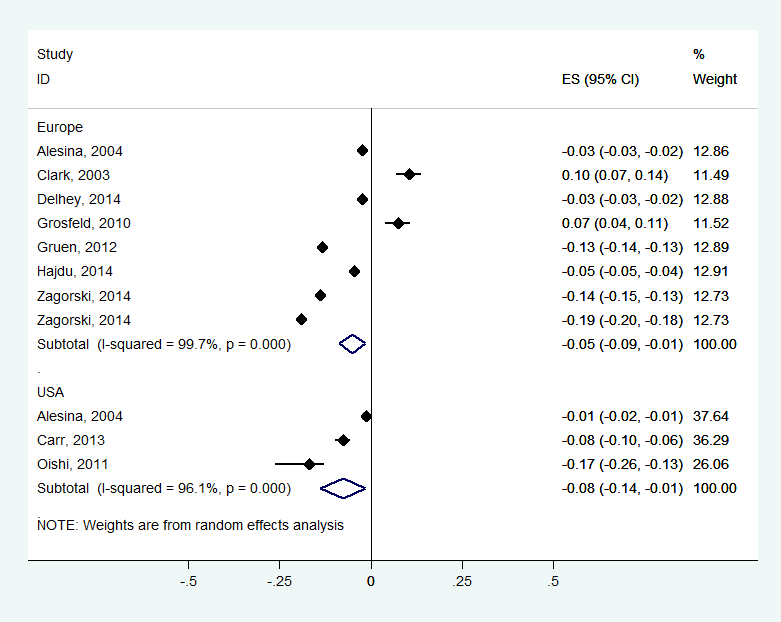


Figure 4. Forest plot displaying meta-analysis of the correlations between income inequality and SWB of sub-group: European countries versus USA.


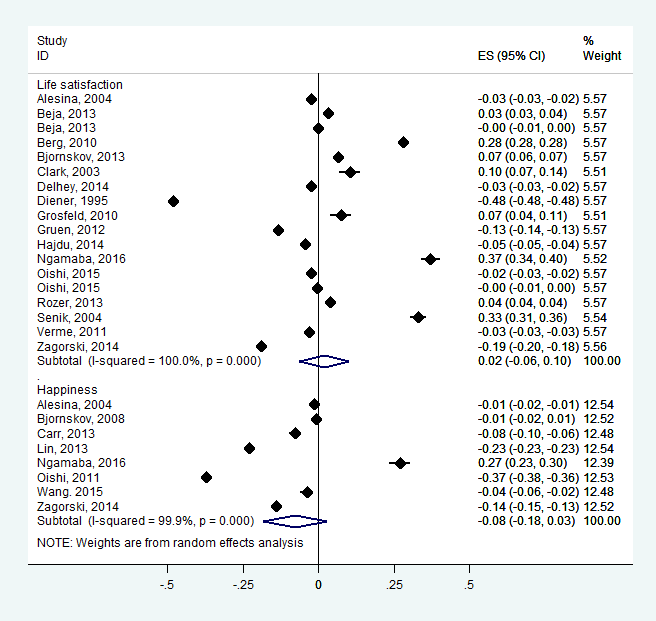


Figure 5. Forest plot displaying meta-analysis of the correlations between income inequality and SWB of sub-group: Life satisfaction versus Happiness


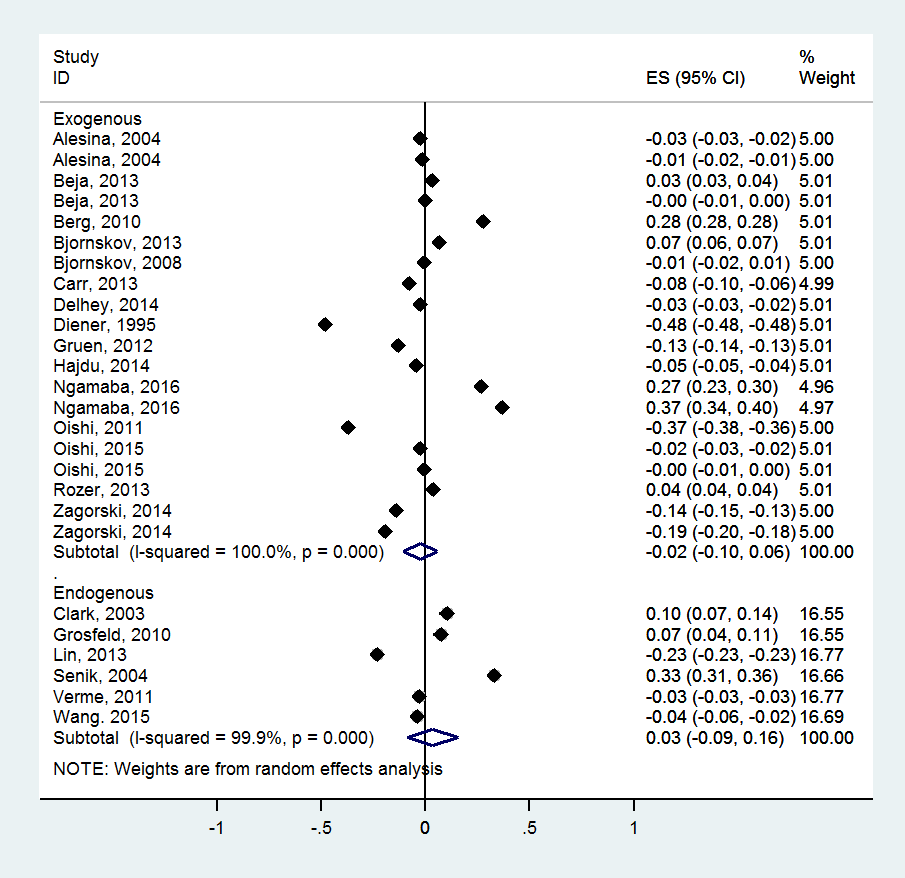


Figure 6. Forest plot displaying meta-analysis of the correlations between income inequality and SWB of sub-group: Exogenous Gini versus Endogenous Gini
